# Synthesis and Health Effects of Phenolic Compounds: A Focus on Tyrosol, Hydroxytyrosol, and 3,4-Dihydroxyacetophenone

**DOI:** 10.3390/antiox14040476

**Published:** 2025-04-16

**Authors:** Wenyu Wang, Lixin Du, Qidong Wei, Mengyao Lu, Dehong Xu, Ya Li

**Affiliations:** School of Pharmacy, Hunan University of Chinese Medicine, Changsha 410208, China; 20233810@stu.hnucm.edu.cn (W.W.); 20223723@stu.hnucm.edu.cn (L.D.); 20233763@stu.hnucm.edu.cn (Q.W.); 20243753@stu.hnucm.edu.cn (M.L.)

**Keywords:** tyrosol, hydroxytyrosol, 3,4-dihydroxyacetophenone, mechanism of action

## Abstract

Tyrosol (Tyr), hydroxytyrosol (TH), and 3,4-Dihydroxyacetophenone (3,4-DHAP) are three phenolic compounds naturally present in plants that have attracted considerable research attention due to their potent antioxidant, anti-inflammatory, anticancer, and cardiovascular protective properties. In recent years, mounting evidence has indicated that these phenolic compounds hold broad potential in both disease prevention and treatment. This paper reviews the chemical structures and synthetic methods of Tyr, HT, and 3,4-DHAP, as well as their multifaceted effects on human health, particularly their roles and mechanisms in antioxidation, anti-inflammation, cardiovascular protection, neuroprotection, and anticancer activity. In addition, this paper explores the future prospects of these compounds and the current challenges associated with their application—such as low bioavailability and long-term safety concerns—and proposes directions for further investigation.

## 1. Introduction

Phenolic compounds are a class of secondary metabolites widely found in plants, characterized by one or more benzene rings bearing one or more hydroxyl (-OH) groups [1]. Tyrosol (Tyr) is a representative phenolic compound; hydroxytyrosol (HT) is its hydroxylated product; while 3,4-Dihydroxyacetophenone (3,4-DHAP) results from the dehydration and hydroxylation of the Tyr isomer 1-(4-hydroxyphenyl)-ethanol [2]. In recent years, Tyr and its hydroxylated derivatives have garnered significant attention for their potential health benefits in preventing and treating chronic diseases, particularly due to crucial advances in antioxidant, anti-inflammatory, anticancer, and cardiovascular protective research [3,4,5,6]. The selection of Tyr, HT, and 3,4-DHAP in this context not only provides mutual validation of their mechanisms of action but also indicates possible directions for further mechanistic exploration.

In everyday diets, phenolic compounds are extensively present in fruits, vegetables, tea, red wine, and olive oil, with an especially pronounced role in the Mediterranean diet [7]. Owing to the abundance of phenolic compounds, notably Tyr and HT, this dietary pattern is believed to exert remarkable cardiovascular protective effects [8]. The most prominent mechanism attributed to phenolic compounds is their antioxidant capability [9], which safeguards cells by scavenging free radicals and reducing oxidative stress [10,11], thereby playing a vital role in chronic disease prevention. Moreover, phenolic compounds can mitigate the occurrence of cardiovascular disease [12], neurodegenerative conditions, and cancers [13] through mechanisms such as modulating inflammatory responses, promoting vasodilation, and inhibiting tumor cell proliferation.

Based on these findings, this paper aims to systematically review phenolic compounds, particularly Tyr, HT, and 3,4-DHAP, focusing on their structures, synthetic methods, and health impacts. Special attention is given to their modes of action in antioxidation, anti-inflammation, cardiovascular protection, neuroprotection, and anticancer effects [14], along with a discussion of their potential clinical applications and directions for future research.

## 2. Methods

A systematic literature search was conducted through the PubMed and Web of Science databases. Original articles on the synthesis methods and health effects of phenolic compounds (including Tyr, HT, and 3,4-DHAP) were identified using the following search terms: Tyr or HT or 3,4-DHAP and synthesis methods or bioactivity or antioxidant or anti-inflammatory or cardioprotective or neuroprotective or osteoprotective or anticancer or antidiabetic or antiobesity or antimicrobial or metabolism or bioavailability, covering the period from 2010 to 2025. These numerous articles were further manually screened for relevance to the research topic. The focus was on new insights related to the synthesis and bioactivity of phenolic compounds, as well as the confirmed effects on human health. Articles published prior to 2010 were carefully selected and incorporated when relevant to the topic.

## 3. Structure and Synthesis of Phenolic Compounds

### 3.1. Structure and Synthesis of Tyrosol

**Figure 1:**
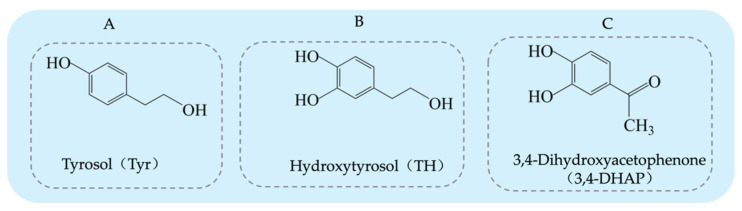
Structural diagrams of tyrosol, hydroxytyrosol, and 3,4-Dihydroxyacetophenone: (**A**) tyrosol structure; (**B**) hydroxytyrosol structure; (**C**) 3,4-Dihydroxyacetophenone structure.

Tyrosol (Tyr) is a typical phenolic compound [15], with a chemical structure consisting of a benzene ring, a hydroxyl group (-OH), and a hydroxyethyl side chain (-CH_2_CH_2_OH) (see Figure 1A). Its chemical formula is C_8_H_10_O_2_ [16]. As an important chemical intermediate, Tyr has wide applications in various industries, including chemistry, pharmaceuticals, agriculture, and fragrances.

**Figure 2:**
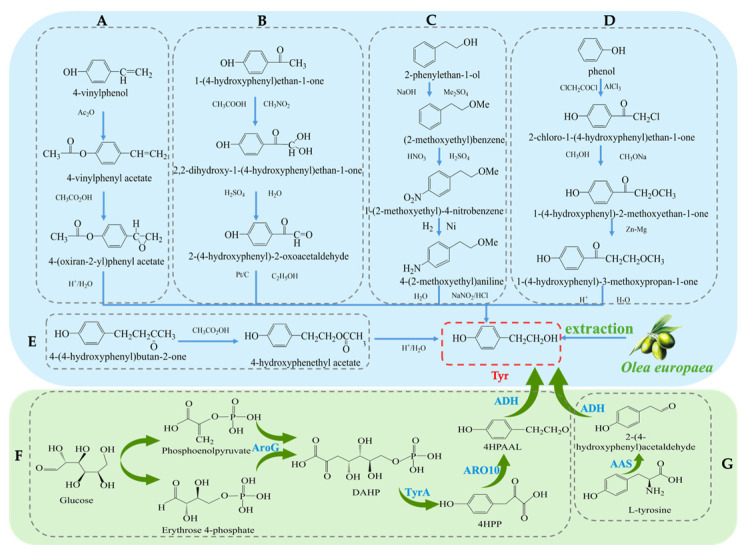
Overall synthesis scheme of tyrosol: (**A**) para-hydroxyphenylethylene synthesis method; (**B**) para-hydroxyacetophenone synthesis method; (**C**) phenylethanol synthesis method; (**D**) phenol synthesis method; (**E**) carboxylate ester synthesis method; (**F**) Glucose-based synthesis method; (**G**) Tyrosine-based synthesis method; AroG: 3-deoxy-D-arabino-heptulosonate-7-phosphate synthase; TyrA: chorismate mutase or prephenate, dehydrogenase; 4HPP: 4-hydroxyphenylpyruvate; ARO10: phenylpyruvate decarboxylase; 4HPAAL: 4- hydroxyphenyacetaldehyde; ADH: alcohol dehydrogenase; AAS: Aromatic Aldehyde Synthase.

The synthesis methods of Tyr have undergone more than a century of development, and currently, there are several synthetic routes available. Traditional methods for obtaining Tyr mainly include plant extraction and chemical synthesis. The plant extraction method involves extraction from the olive plant (*Olea europaea*) [17]. However, plant extraction faces challenges such as long plant growth cycles, low yields, and high raw material costs, which limit its widespread application in industrial production. To overcome the limitations of plant extraction, chemical synthesis methods have been widely applied in the production of Tyr. Several chemical synthesis strategies have been developed to achieve efficient Tyr synthesis [18], each with its own characteristics. Firstly, the synthesis method using para-hydroxyphenylethylene (see Figure 2A) has gained attention due to its simplicity in operation, achieving a high yield of 96% and purity of 99% during the synthesis process [19]. From a technical perspective, this method shows considerable potential for application; however, the high cost of raw materials remains a major bottleneck to its large-scale industrial adoption. Secondly, the para-hydroxyacetophenone method [20] (see Figure 2B) is praised for its simple steps, but the first reaction step requires a temperature of −30 °C, which not only increases equipment and energy costs but also affects the universality of the process to some extent. Considering economic benefits and environmental friendliness, this method still demonstrates competitiveness in certain special applications. Another method uses phenylethanol [21] as the starting material (see Figure 2C), with the basic process including hydroxyl protection, followed by nitration, reduction, diazotization, and hydrolysis, ultimately yielding Tyr with an overall yield of approximately 70%. Despite the numerous steps involved, this route has advantages in terms of raw material availability and operational processes. Additionally, there is a biosynthetic route using phenol [22] as the starting material (see Figure 2D), which involves steps such as chloroacetylation, etherification, Clemmensen reduction, and hydrolysis, efficiently producing the target product. Lastly, the hydrolysis of carboxylate esters [23] (see Figure 2E) is also a method of interest due to its simplicity, but like other methods, environmental concerns still need attention. Overall, although chemical synthesis methods have distinct advantages in improving the yield and purity of Tyr, some reagents and process conditions used in these reactions may still have negative environmental impacts. Future research should focus on optimizing the greening of the process and reducing costs.

In recent years, with the development of biotechnology, biosynthesis has emerged as a new direction for obtaining Tyr. These methods not only offer high efficiency and environmental benefits but also effectively reduce production costs [24]. Common biosynthetic pathways include biosynthesis using *Escherichia coli*, where a dual-plasmid system carrying the tyrosine hydroxylase and decarboxylase genes is constructed. The process (see Figure 2G) involves converting 10 mM tyrosine to 9.56 ± 0.64 mM Tyr at 45 °C with a conversion efficiency exceeding 95% [25]. Another study utilized the overexpression of phenylpyruvate decarboxylase (ARO10) in Saccharomyces cerevisiae to construct a recombinant *E. coli* strain. The process (see Figure 2F) synthesizes Tyr from glucose and optimizes the induction conditions, resulting in an increase in Tyr yield to 9.53 mM [26]. In summary, although traditional plant extraction and chemical synthesis methods remain important routes for Tyr production, biosynthesis technologies are gradually becoming the mainstream method for Tyr production due to their high efficiency, environmental friendliness, low cost, and adjustability in the production process.

### 3.2. Structure and Synthesis of Hydroxytyrosol

**Figure 3:**
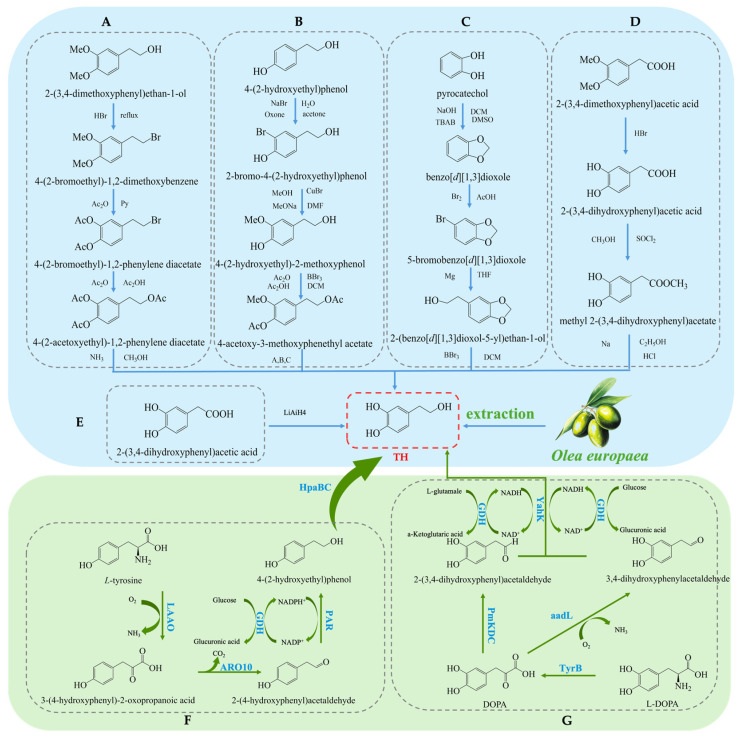
Overall synthesis scheme of hydroxytyrosol: (**A**) synthesis method of 3,4-dimethoxyphenylethanol; (**B**) synthesis method of tyrosol; (**C**) synthesis method of ortho-hydroquinone; (**D**) synthesis method of 3,4-dimethoxyphenylacetic acid; (**E**) synthesis method of 3,4-dihydroxyphenylacetic acid; (**F**) glucose-based synthesis method; (**G**) tyrosine-based synthesis method; aadL: L-amino acid dehydrogenase; KAD: α-ketoacid decarboxylase; TyrB: aromatic amino acid aminotransferase; GDH: L-glutamate dehydrogenase; PmKDC: α-ketoacid decarboxylase; YahK: Aldehyde Reductase; HpaBC: 4-hydroxyphenylacetate 3-Monooxygenase; LAAO: L-amino acid oxidase; ARO10: phenylpyruvate decarboxylase; PAR: Phenylacetaldehyde Reductase.

Hydroxytyrosol (TH) is a natural phenolic compound widely found in olive oil and its by-products [27,28]. Its chemical structure consists of a benzene ring, two hydroxyl groups (both located on the benzene ring), and a hydroxyethyl side chain (-CH₂CH₂OH) (see Figure 1B). Compared to Tyr, TH has an additional hydroxyl group on the benzene ring [29]. The main methods for obtaining TH include plant extraction, chemical synthesis, and biosynthesis. Each method has its advantages and drawbacks. Plant extraction yields relatively low amounts, chemical synthesis is more complex, and biosynthesis has been receiving increasing attention due to its efficiency and environmental friendliness. The plant extraction method involves extracting TH from the olive plant (*Olea europaea*) [30], but this method is often limited by low yield and poor extraction efficiency, and the process may require large amounts of solvents. On the other hand, chemical synthesis methods for TH have become a research hotspot, with various synthetic routes reported in the literature, each with its own strengths and weaknesses. Firstly, the synthesis method using 3,4-dimethoxyphenylethanol as the starting material (see Figure 3A) can produce the target product, but the yield of the final deprotection reaction is only 10%, and the overall steps are numerous, leading to high costs [31]. Secondly, the route using Tyr as the starting material (see Figure 3B) requires the introduction of a hydroxyl group onto the benzene ring, which involves hydroxyl protection and deprotection steps and requires complex reagents such as lithium aluminum hydride and boron tribromide, making it less commonly used in both laboratory and industrial production. Similarly, the synthesis route based on ortho-hydroquinone (see Figure 3C) involves many reaction steps and the use of the highly dangerous boron tribromide, which limits its practical application due to the intensity of the reactions involved [32]. Additionally, the route using 3,4-dimethoxyphenylacetic acid as the starting material (see Figure 3D) can yield TH but involves the use of highly corrosive hydrogen bromide and highly flammable and explosive sodium metal, which presents significant safety hazards [33]. A method using 3,4-dihydroxyphenylacetic acid as the starting material, with lithium aluminum hydride as the reducing agent, directly synthesizes TH in a single reaction step (see Figure 3E). This significant simplification of the reaction steps and the substantial increase in yield present a safer and more efficient route for the synthesis and industrial production of TH [34].

In recent years, biosynthesis methods have gained increasing attention due to their high efficiency and environmental advantages. Researchers have significantly improved TH production by optimizing microorganisms and enzymatic engineering [35]. On one hand, utilizing L-tyrosine as a substrate and employing a modular cascade reaction has achieved the conversion to TH [36], as shown in (Figure 3F). For example, an enzyme cascade involving HpaBC from *Escherichia coli*, L-amino acid deaminase (LAAD) from *Pseudomonas putida*, ARO10 from *Saccharomyces cerevisiae*, and PAR from tomato exhibited high catalytic activity and efficiently synthesized TH. Under 50 mM L-tyrosine conditions, a TH yield of 32.35 mM was obtained [37]. On the other hand, researchers constructed a pathway for converting L-3,4-dihydroxyphenylalanine (L-DOPA) to TH in *E. coli* by co-expressing aromatic amino acid aminotransferase (TyrB), α-ketoacid decarboxylase (PmKDC), YahK, and L-glutamate dehydrogenase (GDH) (see Figure 3G). This system successfully synthesized 36.33 mM of TH [38,39]. This co-expression system not only improved the substrate conversion rate but also significantly increased the yield. Furthermore, by co-expressing L-amino acid dehydrogenase (aadL), YahK, and GDH, the substrate conversion rate was further increased to 89.3% [40]. This multi-enzyme co-expression system, compared to traditional chemical synthesis methods, offers not only higher yields and fewer by-products but also a more environmentally friendly production process.

### 3.3. Structure and Synthesis of 3,4-Dihydroxyacetophenone

3,4-Dihydroxyphenylacetone (3,4-DHAP) is a phenolic compound found in *Ilex cornuta*. sylvestris, with a molecular structure comprising a benzene ring attached to two hydroxyl groups, as well as an acetyl group (-COCH_3_) (see Figure 1C). Its structure is relatively complex, and it possesses potential biological activity. The chemical formula is C_8_H_8_O_3_. It has significant antioxidant, anti-inflammatory, and antitumor effects [41].

**Figure 4:**
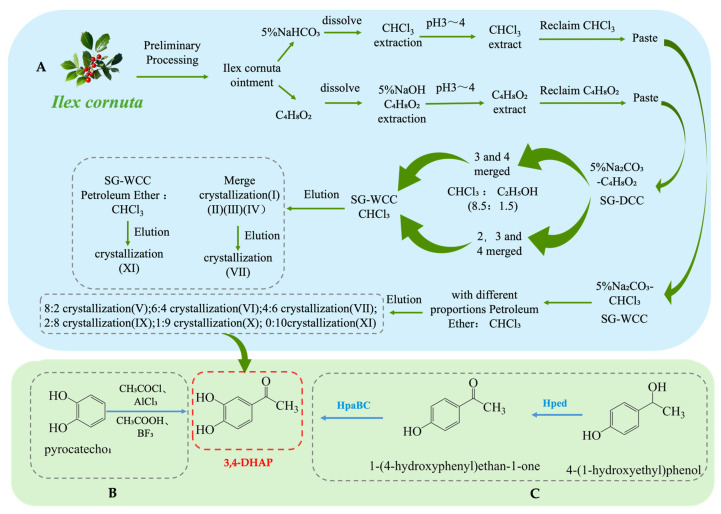
Overall synthesis scheme of 3,4-Dihydroxyacetophenone: (**A**) plant extraction; (**B**) chemical synthesis; (**C**) biosynthesis; Hped: 1-(4-hydroxyphenyl)-ethanol dehydrogenase; HpaBC: 4-hydroxy phenylacetate 3-hydroxylase; SG-WCC: Silica Gel Wet Column Chromatography; SG-DCC: Silica Gel Dry Column Chromatography.

As one of the main active ingredients in *Ilex cornuta*. sylvestris, the extraction methods for 3,4-DHAP mainly include traditional plant extraction, chemical synthesis, and biosynthesis. Firstly, 3,4-DHAP can be extracted from *Ilex cornuta*. sylvestris using traditional plant extraction methods [42] (see Figure 4A). This method is relatively complex, involving multiple steps such as dissolution, extraction, separation, chromatography, elution, and crystallization, and it has the limitation of low extraction efficiency. Additionally, studies have found that 3,4-DHAP can also be isolated from the ether fraction of water extracts from *Spruce needles* [43]. Moreover, 3,4-DHAP can also be synthesized chemically (see Figure 4B). The first chemical synthesis method uses acetyl chloride (CH_3_COCl) as the acetylating reagent, anhydrous aluminum chloride (AlCl_3_) as the catalyst, and carbon disulfide (CS_2_) as the reaction medium, with the reaction carried out by heating and refluxing. However, the yield of this method is relatively low (only 37%), which may be due to suboptimal reaction conditions [44]. The second method uses acetic acid (CH_3_COOH) as the acetylating reagent, boron trifluoride (BF_3_) as the catalyst, and ether (C_4_H_10_O) as the reaction medium. The reaction is conducted at 80 °C for 4 h, achieving a yield of 66.7%, which is higher than the first method, and the reaction conditions are milder [45].

In addition, biosynthesis studies of 3,4-DHAP have also been reported. A whole-cell biotransformation method has been developed by constructing a recombinant *E. coli* strain containing the Hped gene (encoding 1-(4-hydroxyphenyl)-ethanol dehydrogenase) and the HpaBC gene (encoding 4-hydroxyphenylacetate 3-hydroxylase) (see Figure 4C). This system converts 1-(4-hydroxyphenyl)-ethanol into 3,4-DHAP, with a maximum yield of 260 mg·L^−1^ and a conversion rate of 17%. Although the conversion rate is relatively low, this method offers a potential green synthesis route and is more environmentally friendly compared to traditional chemical synthesis methods [46].

## 4. Health Effects of Phenolic Compounds

### 4.1. Antioxidant Activity

**Figure 5:**
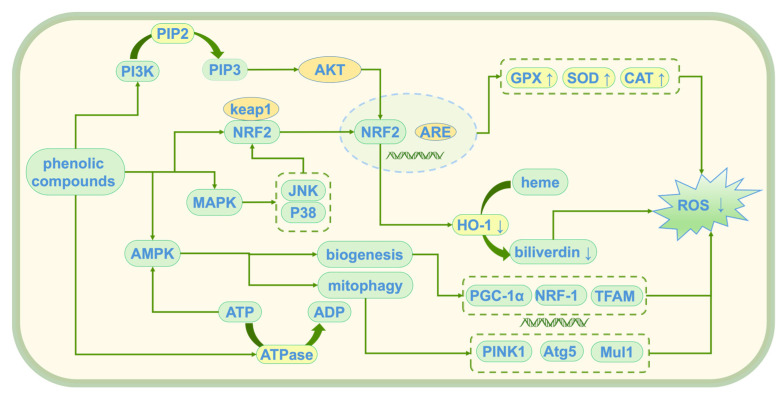
Antioxidant mechanism of phenolic compounds; ↑: promote generation; ↓: Inhibit generation; PI3K: Phosphoinositide 3-Kinase; PIP2: Phosphatidylinositol 4,5-bisphosphate; PIP3: Phosphatidylinositol (3,4,5)-trisphosphate; Akt: Protein Kinase B (PKB); Keap1: Kelch-like ECH-associated Protein 1; Nrf2: Nuclear Factor Erythroid 2-Related Factor 2; ARE: antioxidant response element; MAPK: mitogen-activated protein kinase; JNK: c-Jun N-terminal Kinase; AMPK: AMP-activated protein kinase; ATP: Adenosine Triphosphate; ADP: Adenosine Diphosphate; ATPase: Adenosine Triphosphatase; HO-1: heme oxygenase-1; ROS: reactive oxygen species; GPX: glutathione peroxidase; SOD: superoxide dismutase; CAT: catalase; PINK1: PTEN-induced putative kinase 1; Atg5: Autophagy-related 5; Mul1: Mitochondrial Ubiquitin Ligase 1; PGC-1α: Peroxisome Proliferator-Activated Receptor Gamma Coactivator 1-alpha; NRF-1: Nuclear Respiratory Factor 1; TFAM: Mitochondrial Transcription Factor A.

Tyr, HT, and 3,4-DHAP, as natural antioxidants, have demonstrated significant antioxidant activity [47]. These compounds alleviate oxidative stress damage induced by reactive oxygen species (ROS) through multiple mechanisms, thereby maintaining cellular homeostasis. Their main mechanisms of action (see Figure 5) include the direct scavenging of ROS, activation of endogenous antioxidant enzymes, inhibition of lipid peroxidation, modulation of oxidative stress-related pathways, and protection of mitochondrial and genomic integrity. Although the antioxidant mechanisms of these compounds are similar, their molecular structural differences result in varying targets and efficiencies. HT, due to its polyphenolic structure, exhibits a stronger free radical scavenging ability than Tyr. In contrast, 3,4-DHAP mainly exerts its antioxidant effects indirectly by modulating the Nrf2/ARE signaling pathway.

#### 4.1.1. Free Radical Scavenging

ROS and other free radicals are widely considered key initiators of cellular damage and various diseases [48]. Tyr reduces cellular damage by directly reacting with superoxide anions, transforming them into stable molecules [49,50,51]. TH further enhances the antioxidant effect, not only scavenging superoxide anions but also neutralizing hydroxyl radicals and other free radicals [52,53]. Additionally, TH can inhibit the chain reaction of peroxy radicals, preventing their diffusion and significantly enhancing antioxidant capacity [54]. The mechanism of 3,4-DHAP is similar to that of TH; it can scavenge superoxide anion and hydroxyl free radicals [55]. When treated with 3,4-DHAP and exposed to high-glucose conditions, HUVEC cells showed a significant reduction in ROS generation [56].

#### 4.1.2. Antioxidant Enzyme Activation

The antioxidant enzyme system is a key defense mechanism against oxidative damage. By activating endogenous antioxidant enzymes, it enhances the cell’s self-repair and defense capabilities, effectively responding to oxidative stress [57]. Tyr enhances the mRNA expression of superoxide dismutase (SOD) or inhibits its degradation, thereby increasing SOD activity and promoting the conversion of superoxide anions to hydrogen peroxide. This hydrogen peroxide is further cleared by glutathione peroxidase (GPx) and catalase (CAT), effectively reducing oxidative damage induced by superoxide anions [58]. TH and 3,4-DHAP enhance SOD activity through similar mechanisms, maintaining cellular redox balance. Additionally, Tyr activates GPx and CAT, accelerating the decomposition of H_2_O_2_, and preventing its accumulation and oxidative damage [59]. TH further enhances GPx and CAT activity, optimizing H_2_O_2_ clearance [60]. 3,4-DHAP also promotes GPx activity, thereby enhancing the cell’s defense against oxidative damage [61].

#### 4.1.3. Inhibition of Lipid Peroxidation

Lipid peroxidation is an oxidative damage process initiated by free radicals, involving the reaction of lipids in the cell membrane with free radicals. The resulting lipid peroxides damage the membrane structure, directly affecting the structure and function of the cell membrane [62]. Reducing lipid peroxidation helps maintain the stability and fluidity of the cell membrane, preventing membrane damage and further protecting cellular integrity [63,64,65]. The oxidative modification of HDL decreases its antioxidant function, further exacerbating lipid peroxidation. Tyr inhibits lipid peroxidation by reducing the oxidative modification of HDL, thereby enhancing HDL function, particularly promoting cholesterol efflux and preventing the generation of lipid peroxides in the cell membrane, thus stabilizing the cell membrane [66]. TH reduces serum cholesterol, total cholesterol, and low-density lipoprotein cholesterol (LDL-C) levels while increasing high-density lipoprotein cholesterol (HDL-C) levels, slowing the lipid peroxidation process [67,68]. Studies have shown that pre-treatment of endothelial cells with TH enhances SOD activity and inhibits lipid peroxidation [69]. 3,4-DHAP reduces lipid peroxidation by decreasing free radicals, thus preventing oxidative damage to the cell membrane and maintaining its normal function [70,71,72].

#### 4.1.4. Modulation of Oxidative Stress Response

Oxidative stress refers to the biological reactions that occur when ROS accumulate beyond the cell’s antioxidant capacity, leading to cellular damage [73]. Studies have shown that Tyr can regulate the Nrf2/ARE signaling pathway, mitogen-activated protein kinase (MAPK) pathways, c-Jun N-terminal kinase (JNK), p38 MAPK, and increase ATP production to enhance the cell’s energy supply, improving the antioxidant stress response and inhibiting H_2_O_2_-induced cell death, thereby reducing oxidative damage [74]. TH not only activates the Nrf2-ARE pathway [75] but also modulates the synthesis and activity of antioxidant enzymes via the PI3K/Akt and MAPK pathways [76]. Notably, TH can reduce the duration of JNK and p38 phosphorylation induced by H_2_O_2_, likely due to its ability to alleviate oxidative stress by influencing intracellular iron homeostasis [77]. Additionally, TH reduces the catalytic effect of metal ions (such as Fe^2^⁺ and Cu^2^⁺) in oxidative reactions by chelating these ions, thus inhibiting metal ion-induced oxidative damage [78]. Studies have shown that 3,4-DHAP modulates the Nrf2/HO-1 pathway, increases the protein and mRNA expression of antioxidant proteins Nrf-2 and HO-1, promotes Nrf2 nuclear translocation, reduces ROS production, and enhances cellular antioxidant defenses [56,79].

#### 4.1.5. Mitochondrial Protection

Mitochondria are the energy factories of cells, and their proper function is crucial for cellular energy metabolism [80]. Oxidative damage affects mitochondrial function, disrupting the cell’s energy supply [81]. Tyr protects mitochondrial function by scavenging free radicals within the mitochondria and reducing oxidative damage to the mitochondrial membrane, thereby ensuring the stability of mitochondrial function and energy metabolism [82]. TH restores energy deficits in Alzheimer’s disease cell models (AD models) by enhancing mitochondrial activity [83]. It also reduces the production of mitochondrial superoxide, thereby protecting mitochondrial function [69]. Studies have shown that TH activates the AMPK signaling pathway; regulates the expression of the PGC-1α, NRF-1, and TFAM genes involved in mitochondrial biogenesis; and promotes mitochondrial function recovery. Additionally, it facilitates mitochondrial autophagy through the activation of PINK1, MUL1, and ATG5, improving mitochondrial function [84] and significantly reducing mitochondrial alterations and oxidative damage [85]. 3,4-DHAP increases Na+, K+, and ATPase activity; reduces peroxide generation; and inhibits ascorbate and ferrous sulfate-induced damage to brain mitochondria and cells [86]. Overall, these antioxidant compounds play an important role in reducing mitochondrial oxidative damage and maintaining energy metabolism stability.

#### 4.1.6. DNA and Protein Protection

Phenolic compounds protect cellular DNA and proteins by reducing oxidative damage, preventing gene mutations, protein denaturation, and loss of cellular function [59]. Both Tyr and TH protect DNA by scavenging ROS, reducing oxidative DNA damage, and preventing gene mutations, thereby maintaining normal protein function [87]. Studies have shown that TH significantly reduces ROS levels in human mammary epithelial cells (MCF10A) and effectively prevents oxidative DNA damage in breast cancer cell lines (such as MCF7 and MDA-MB-231) [88]. TH can effectively reduce DNA breakage, protecting DNA from H_2_O_2_-induced DNA fragmentation [89]. 3,4-DHAP promotes the formation of autophagosomes, which indirectly reduces DNA damage by clearing damaged mitochondria, thus protecting DNA from oxidative stress and maintaining protein structure and function [56].

### 4.2. Anti-Inflammatory Effects

**Figure 6:**
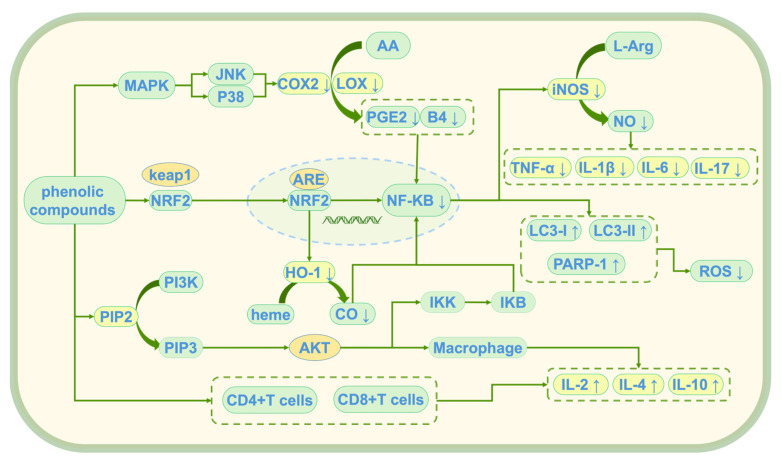
Anti-inflammatory mechanism of phenolic compounds; ↑: promote generation; ↓: Inhibit generation; AA: Arachidonic Acid; L-Arg: L-Arginine; COX2: Cyclooxygenase-2; PGE2: prostaglandin E2; B4: leukotriene B4 (LTB4); iNOS: Inducible Nitric Oxide Synthase; NO: nitric oxide; TNF-α: Tumor Necrosis Factor Alpha; IL-1β: Interleukin-1 Beta; IL-6: Interleukin-6; LC3-I/LC3-II: Microtubule-Associated Protein 1 Light Chain 3 (LC3); PARP-1: Poly (ADP-Ribose) Polymerase-1; ROS: reactive oxygen species; MAPK: mitogen-activated protein kinase; JNK: c-Jun N-terminal Kinase; p38: p38 MAPK; Keap1: Kelch-like ECH-associated Protein 1; Nrf2: Nuclear Factor Erythroid 2-Related Factor 2; ARE: antioxidant response element; NF-κB: Nuclear Factor Kappa B; HO-1: heme oxygenase-1; heme: heme; CO: Carbon Monoxide; PI3K: Phosphoinositide 3-Kinase; PIP2: Phosphatidylinositol 4,5-Bisphosphate; PIP3vPhosphatidylinositol (3,4,5)-Trisphosphate; Akt: Protein Kinase B (PKB); IKK: IκB Kinase; IκB: Inhibitor of NF-κB; IL-2: Interleukin-2; IL-4: Interleukin-4; IL-10: Interleukin-10; LOX: lipoxygenase.

Phenolic compounds exert their anti-inflammatory effects through multiple mechanisms, collaboratively alleviating chronic inflammation induced by oxidative stress [90]. These anti-inflammatory effects are mainly achieved through three key mechanisms: the inhibition of inflammation initiation triggered by oxidative stress, modulation of the balance between pro-inflammatory and anti-inflammatory factors, and regulation of immune cell function [91]. Through these mechanisms (see Figure 6), they not only provide powerful antioxidant protection for cells but also reduce inflammatory damage by regulating immune responses, offering new therapeutic strategies for the prevention and treatment of chronic inflammation-related diseases induced by oxidative stress.

#### 4.2.1. Inhibition of Inflammation Initiation Triggered by Oxidative Stress

Scavenging free radicals can reduce oxidative stress, thereby inhibiting the inflammation response initiated by oxidative stress [92]. Tyr reduces inflammation initiation by inhibiting the signaling of CD14 (a TLR4 co-receptor) downstream of the MAPK pathway, thereby attenuating the amplification of inflammation signals [93], thus reducing oxidative stress-induced inflammation [74]. TH activates the Nrf2/ARE and PI3K/Akt pathways [94], inhibiting the expression of COX-2, iNOS, NLRP3 inflammasome, ADAMTS-4, and MMP-13, thereby alleviating inflammation induced by ROS [95]. 3,4-DHAP modulates the JNK/Nrf2 pathway, upregulating eNOS-NO signaling, which synergistically enhances endothelial protection and inhibits the vicious cycle between oxidative stress and inflammation [64,96,97,98].

#### 4.2.2. Regulation of the Balance Between Pro-Inflammatory and Anti-Inflammatory Factors

The NF-κB pathway plays a central role in the inflammatory response. Its excessive activation can lead to the release of pro-inflammatory cytokines such as TNF-α, IL-1β, and IL-6, which exacerbates inflammation [99]. The inhibition of NF-κB activation can reduce the release of pro-inflammatory cytokines, thus mitigating the inflammatory response [100,101]. In a colitis-induced model, Tyr reduces inflammation by activating the MAPK pathway and regulating the gene expression of IL-6, COX-2, and NF-κB [102]. At concentrations ranging from 0.150 to 300 μmol/L, Tyr also inhibits the release of inflammatory cytokines TNF-α, IL-1β, and IL-6 [103,104,105]. TH modulates the Nrf2/ARE and PI3K/Akt pathways [106], inhibits IκB kinase (IKK) activity, and prevents NF-κB nuclear translocation, thus reducing the transcription of pro-inflammatory cytokines such as TNF-α and IL-1β [107,108]. It also inhibits the expression of pro-inflammatory iNOS and COX-2, and the activation of granulocytes and monocytes [109,110,111,112]. In colitis, TH reduces colitis biomarkers such as MPO and pro-inflammatory cytokine IL-6, thereby mitigating inflammation [113]. TH also exerts combined antioxidant and anti-inflammatory effects by downregulating pro-inflammatory cytokines and upregulating the anti-inflammatory gene Bcl2 [114]. In a study evaluating the anti-inflammatory ability and potential mechanisms of 3,4-DHAP, it was found that the compound increases Nrf2 and HO-1 protein and mRNA expression, promotes Nrf2 nuclear translocation, and enhances autophagosome formation. This significantly increases the expression of LC3 II/LC3 I, a major modification of microtubule-associated protein 1, and PARP1 proteins, promoting autophagy and DNA damage repair, while significantly reducing the expression of COX-2 and IL-17, thus promoting inflammation resolution [56].

#### 4.2.3. Regulation of Immune Cell Function

Immune cells (macrophages, T cells, and B cells) play a crucial role in the inflammatory response. Overactive immune cells release large amounts of inflammatory cytokines, causing tissue damage. Reducing the excessive activation of immune cells can alleviate inflammation induced by immune responses. Tyr not only inhibits the production of ROS by activated macrophages but also directly scavenges ROS, inhibiting the activity of phospholipase A2 (PLA2), and reducing the release of AA from membrane phospholipids, thus blocking prostaglandin E2 (PGE2) synthesis and reducing the expression of COX-2 and iNOS. This decreases the synthesis of PGE2, leukotriene B4 (LTB4), and nitric oxide (NO) [115,116]. Tyr also modulates the function of macrophages, T cells, and B cells by activating the MAPK pathway, reducing the excessive activation of immune cells, and alleviating inflammation induced by immune responses [108]. TH increases the expression of CD^4+^ T cells and CD^8+^ T cells; promotes the mRNA expression of IL-2, IL-4, and IL-10; and enhances the activity of SOD and GSH-Px, while reducing the MDA levels, thereby alleviating inflammation [82]. 3,4-DHAP promotes the expression of HO-1 and CO in macrophages [117,118], significantly upregulates the mRNA and protein expression of soluble Toll-like receptor 4 (sTLR4), and reduces TNF-α secretion [119,120], thus regulating macrophage apoptosis and promoting the resolution of inflammation [121,122].

### 4.3. Cardiovascular Protective Effects

Tyr, HT, and 3,4-DHAP are natural phenolic compounds found in olives, and they exhibit significant cardiovascular protective effects. Through various mechanisms, they positively influence cardiovascular health, including antioxidant, anti-inflammatory, lipid-lowering, and vascular function-enhancing effects. These compounds can effectively alleviate the occurrence and development of cardiovascular diseases. The following summarizes their roles in different cardiovascular diseases.

#### 4.3.1. Protection Against Atherosclerosis

Atherosclerosis is a major cause of cardiovascular diseases, often initiated by oxidized LDL, inflammatory responses, and lipid accumulation. Tyr alleviates oxidative damage by enhancing antioxidant enzyme activity and reduces the deposition of oxidized LDL in blood vessel walls [123]. It inhibits the production of leukotriene B4 (LTB4), which affects endothelial function [124], and also suppresses the degradation of IκBα, thereby inhibiting NF-κB activity and further reducing inflammation [125]. TH prevents the progression of atherosclerosis by activating the Nrf2/ARE signaling pathway, which reduces LDL oxidation [27]. It also regulates vascular inflammation by modulating the autophagy of vascular adventitial fibroblasts (VAF) through the SIRT1-mediated Akt/mTOR pathway, thereby alleviating inflammation [126]. TH metabolites bind to HDL, providing local antioxidant protection and preventing the oxidative modification of HDL proteins and apolipoprotein A-I (ApoA-I), thus reducing oxidative stress and inflammation and benefiting atherosclerosis [127]. 3,4-DHAP reduces the expression levels of TLR4, 5-lipoxygenase (5-LOX), and LTB4 in plaque macrophages while decreasing the expression of vascular cell adhesion molecule-1 (VCAM-1) in plaques, thereby slowing the progression of atherosclerosis and stabilizing plaques [128,129,130,131].

#### 4.3.2. Protection Against Hypertension

Hypertension is an important risk factor for cardiovascular diseases. Improving vascular function, alleviating oxidative damage to the vascular endothelium, and reducing the proliferation of vascular smooth muscle cells can lower blood pressure. Tyr significantly increases the secretion of vascular endothelial growth factor-A (VEGF-A) and platelet-derived growth factor-BB (PDGF-BB) in skeletal muscle cells, enhancing the proliferation and migration of endothelial cells and smooth muscle cells (the two cell types responsible for blood vessel formation) [132]. Tyr also inhibits the development of TH syndrome, improving oxygen transport capacity and increasing cortical microvascular density [133]. TH lowers blood pressure by improving vascular relaxation responses [134]. 3,4-DHAP improves vascular health and reduces hypertension progression by enhancing antioxidant and anti-inflammatory effects [135], modulating the ratio of TXA2 to prostacyclin (PGI2) [136], and inhibiting the expression of hypoxia-inducible NF-κB and COX2 [137]. It also reduces the hypoxia-induced Ca^2+^ increase in smooth muscle cells (PASMCs) [138].

#### 4.3.3. Protection Against Coronary Artery Disease

Coronary artery disease is caused by the blockage or hardening of coronary arteries. Tyr improves blood lipid metabolism by increasing HDL cholesterol levels [139], reducing the oxidation of LDL cholesterol [140], decreasing plaque formation in heart vessels, and lowering the risk of coronary artery disease [141]. TH regulates endoplasmic reticulum (ER) stress in the human hepatocellular carcinoma cell line HepG2 and prevents the oxidation of LDL [142]. 3,4-DHAP regulates the COX and NO/endothelium-dependent relaxing factor (EDRF) signaling pathways, reducing vascular tension and alleviating the risk of blockage, thus slowing the progression of coronary artery disease [143]. It also increases the ratio of cyclic adenosine monophosphate (cAMP) to cyclic guanosine monophosphate (cGMP) in the blood [144], modulates Ca^2+^ levels [138], effectively reducing pulmonary artery pressure, pulmonary vascular resistance, and systemic vascular resistance; increasing cardiac output; and slowing the heart rate [145].

#### 4.3.4. Prevention of Thrombosis

Platelet aggregation is the first step in thrombosis, regulated by a series of cytokines. When the blood vessel wall is damaged, the coagulation system is activated, causing the release of AA from platelet membranes, which in turn, under the catalysis of COX, forms TXA2 and promotes platelet aggregation. Early studies have shown that the addition of TH in the diet of type 1 diabetic patients significantly reduced the content of thromboxane B2 (the inactive form of TXA2) in their blood samples, suggesting that TH may reduce platelet aggregation by inhibiting TXA2 production [146]. Additionally, TH inhibits COX activity and expression [147] and may also reduce platelet aggregation through the inhibition of the cyclic adenosine monophosphate–phosphodiesterase (PDE) pathway [148]. Tyr inhibits the production of ROS by activated macrophages, suppresses AA release and COX-2 and iNOS expression, and reduces the synthesis of prostaglandin E2 (PGE2), leukotriene B4 (LTB4), and NO [115,116]. Reducing the production of pro-thrombotic prostaglandins and thromboxanes or inhibiting excessive COX2 activity is an effective approach for thrombosis prevention and treatment. 3,4-DHAP significantly reduces the expression of COX2 and prostaglandin PGE2 while increasing the expression of prostaglandin PGJ2 [149], thus inhibiting the production of TXA2 and B2 (TXB2) [150,151].

### 4.4. Prevention of Neurodegenerative Diseases

Phenolic compounds, such as TH, have increasingly been studied in recent years for their potential in preventing neurodegenerative diseases [152]. These compounds exhibit significant antioxidant, anti-inflammatory, and anti-apoptotic effects, playing a key role in improving neuronal health [153] and slowing the progression of neurodegenerative diseases. Neurodegenerative diseases, including Alzheimer’s disease (AD) [154], Parkinson’s disease (PD), and Huntington’s disease (HD), are closely associated with factors such as oxidative stress, inflammation, and neuronal cell death [155].

#### 4.4.1. Neuroprotective Effects

Oxidative stress and chronic neuroinflammation are key factors in the development of neurodegenerative diseases such as Alzheimer’s disease and Parkinson’s disease [156]. Excessive ROS damages neuronal membranes, proteins, and DNA, ultimately leading to cell death [157]. High levels of oxidative stress and nitrosative stress compromise the integrity and function of brain tissue, particularly during aging. TH has powerful free radical scavenging properties and induces antioxidant enzymes, making it beneficial for treating some neurodegenerative diseases, such as Parkinson’s disease [158]. TH is also highly effective in inhibiting the formation of toxic amyloid proteins and reversing the toxic effects of insulin amyloid aggregates in neuroblastoma cell lines [159]. By activating the Nrf2/ARE pathway, TH reduces the accumulation of free radicals and ROS, protecting neurons from excessive oxidative stress [160]. Furthermore, it inhibits inflammation pathways such as NF-κB and COX-2, reduces the activation of microglial cells, and alleviates neuroinflammatory damage caused by chronic inflammation [161]. 3,4-DHAP increases the expression of Nrf2 and HO-1 proteins and mRNA, promotes Nrf2 nuclear translocation, and enhances autophagic activity, thereby reducing ROS production and indirectly protecting neurons [56].

#### 4.4.2. Anti-Apoptotic Effects on Neurons

Apoptosis (programmed cell death) of neurons is a major hallmark of the progression of neurodegenerative diseases. Oxidative stress and inflammation activate apoptosis-related signaling pathways, leading to neuronal cell death. Tyr regulates the expression of apoptosis-related proteins such as p53 and Bcl-2, inhibits apoptotic signaling, and promotes neuronal cell survival, thereby slowing the progression of neurodegenerative diseases [162]. TH improves neuronal damage in AD by regulating mitochondrial oxidative stress, neuronal inflammation, and cell apoptosis, without affecting amyloid precursor protein (AβPP) processing [163]. It also increases intracellular ROS levels, p53, and γH2AX (phosphorylated H2AX) expression, and reduces AKT expression, significantly decreasing melanoma cell viability and inducing apoptosis [27].

#### 4.4.3. Regulation of Protein Aggregation

Abnormal protein aggregation and deposition (such as β-amyloid and α-synuclein) are major causes of neuronal damage in Alzheimer’s disease and Parkinson’s disease. Tyr inhibits the enhancement of 4-hydroxy-2-nonenal-induced immune response in the hippocampal CA3 region of AD mice [164] and provides molecular insight into its protective signaling effects on endoplasmic reticulum stress-induced β-cell death [165]. Tyr effectively reduces α-synuclein inclusions, thus delaying the degeneration of α-synuclein-dependent dopaminergic neurons in vivo. Tyr treatment also reduces ROS levels and promotes the expression of specific molecular chaperones and antioxidant enzymes, showing potential as a nutritional supplement targeting key pathogenic factors in Parkinson’s disease [166]. Protein aggregation is the basis of many human diseases. Studies have shown that TH effectively inhibits the aggregation of egg white lysozyme fibrils, suggesting its potential in treating Alzheimer’s disease, Parkinson’s disease, and related conditions [167].

### 4.5. Anticancer Effects

In the past decade, phenolic compounds such as TH have been extensively studied for their significant anticancer effects. Numerous in vitro and in vivo studies have shown that these phenolic compounds inhibit the proliferation of various cancer cell types, induce apoptosis, suppress tumor metastasis, and regulate antioxidant and anti-inflammatory pathways, exhibiting strong anticancer bioactivity [27,168].

#### 4.5.1. Prevention of Cancer Cells

Cancer development is often closely related to oxidative stress and chronic inflammation. Tyr not only reduces oxidative stress-induced cellular damage by activating antioxidant enzymes to scavenge ROS but also alleviates chronic inflammation in the tumor microenvironment by inhibiting the activation of the HIF-1α/NF-κB signaling pathway, thereby reducing the release of inflammatory factors such as TNF-α and IL-6, thus inhibiting cancer initiation and progression [169]. Moreover, Tyr inhibits the PI3K/Akt/mTOR/S6K signaling pathway and reduces the expression of hypoxia-inducible factor 1α (HIF-1α) and its target genes. Additionally, Tyr binds weakly to the cytoplasmic transcription factor AhR and even decreases its transcriptional activity, thereby exerting control over tumor progression in hypoxic conditions [170]. TH downregulates ROS levels and HIF-1α expression in MCF-7 breast cancer cells and can bind to the aryl hydrocarbon receptor (AhR) at high concentrations. It reduces the expression of NF-κB and COX-2, mitigating cell mutations and tumor growth caused by chronic inflammation. Furthermore, TH, when combined with paclitaxel, improves oxidative stress status, reduces chemotherapy side effects, and enhances the antitumor effects of paclitaxel, improving the overall health of patients [171].

#### 4.5.2. Regulation of Cell Apoptosis

Apoptosis is a critical mechanism to prevent carcinogenesis by eliminating abnormally proliferating cells to maintain cellular homeostasis. By regulating apoptosis-related signaling pathways, apoptosis in cancer cells can be promoted, and tumor growth can be inhibited. Tyr induces apoptosis in cancer cells by regulating the expression of apoptosis-related proteins such as p53 and Bcl-2, inhibiting anti-apoptotic signals, and promoting cell survival, thereby reducing tumor growth. TH can also prevent UVB-induced apoptosis in HaCaT cell lines [172]. It reduces the viability of human prostate cancer cells (PC-3 and DU145) through ROS-mediated apoptosis and mitochondrial dysfunction, preventing prostate cancer, and thus providing a foundation for its potential as an anticancer agent [173]. In recent years, extensive research has shown that the antitumor activity of TH is mainly associated with its ability to scavenge ROS and regulate the antioxidant system [174]. Studies have shown that TH inhibits the activation of the AKT and NF-κB pathways, induces G1/S cell cycle arrest, and inhibits the expression of cyclin D1/E and CDK2/4, ultimately reducing androgen receptor and prostate-specific antigen (PSA) levels, supporting its potential application in prostate cancer treatment [175,176]. Cholangiocarcinoma, a digestive tumor with high mortality due to difficulties in early diagnosis and resistance to chemotherapy, has also shown potential anticancer effects from TH. It induces cell cycle arrest and apoptosis in both in vitro and in vivo models, suppressing the proliferation of the TFK-1, KMBC, and GBS-SD cell lines [177]. High doses of TH reduce thyroid cancer cell viability, downregulate cyclin D1 expression, and upregulate the key regulator P21, with Annexin V-P1 staining and DNA ladder assays showing its pro-apoptotic effects on papillary and follicular thyroid cancer cells, further confirming its ability to induce apoptosis and reduce thyroid cancer cell viability [178]. 3,4-DHAP promotes apoptosis by inhibiting NF-κB activation and TNF-α autocrine production, significantly enhancing cell death [179]. Additionally, 3,4-DHAP increases heme oxygenase-1 (HO-1) and CO levels in macrophages [117,118], significantly upregulates soluble Toll-like receptor 4 (sTLR4) mRNA and protein expression, and reduces TNF-α secretion, thus contributing to the regulation of cell apoptosis [119,120].

#### 4.5.3. Inhibition of Cancer Cell Proliferation

The excessive proliferation of cancer cells is a key hallmark of cancer progression. By regulating signaling pathways, cancer cell proliferation can be suppressed, reducing tumor cell division and growth. The MAPK and PI3K/Akt pathways are closely related to cell growth, proliferation, and survival. Tyr significantly reduces cancer cell proliferation by inhibiting these pathways, thereby slowing tumor expansion [180]. Xue Lijun et al. suggested that Tyr induces the expression of phase II detoxifying enzyme NAD(P)H/quinone oxidoreductase-1 (NQO1) in liver cancer cells via the antioxidant response element (ARE), thereby inhibiting the proliferation of human liver cancer SMMC-7721 cells [181,182]. Studies have shown that TH inhibits the expression of chemokine CCL5 in normal human fibroblasts (NHF), thereby blocking the activation of the ERK1/2-cyclin D1 pathway, which suppresses its proliferative effect on MB231 cells. Compared to paclitaxel alone, the combined treatment significantly reduced tumor volume and improved the oxidative status without affecting chemotherapy efficacy. Furthermore, TH is an effective adjunct in paclitaxel treatment for breast cancer. The combination with TH ensures minimal oxidative damage caused by chemotherapy agents, potentially improving patient health [183]. In addition, when used in combination with cetuximab, TH downregulates epidermal growth factor receptor (EGFR) expression, thereby inhibiting colon cancer cell proliferation. The synergistic effect of this combination reduces the required drug concentration by about 10 times compared to monotherapy and enhances the chemotherapy effect [184]. TH inhibits the PI3K/Akt pathway and activates the MAPK pathway, regulating the expression of cell cycle-related proteins (such as Cyclin D1 and CDK4), slowing cancer cell proliferation, and preventing uncontrolled cell growth [27]. It also alters several genes related to cell proliferation, apoptosis, and the Wnt signaling pathway, promoting high expression of the secreted Wnt ligand-related protein 4 (Sfrp4), thus inhibiting breast tumor growth and cell proliferation [185]. Moreover, studies have found that 3,4-DHAP significantly reduces the expression of focal adhesion kinase (FAK) in human pulmonary vascular smooth muscle cells while upregulating caspase-3 expression, thereby inhibiting pathological proliferation [186].

### 4.6. Liver Protection and Metabolic Regulation

Tyr, HT, and 3,4-DHAP are natural phenolic compounds that have been shown in numerous studies to protect the liver from damage through various mechanisms, including antioxidant, anti-inflammatory, anti-fibrotic effects, and the regulation of lipid metabolism. The following is a summary of their mechanisms of action in liver protection.

#### 4.6.1. Hepatocyte Protection

The liver is the body’s primary detoxifying organ. Prolonged exposure to harmful substances such as drugs, toxins, and alcohol can induce oxidative stress, leading to liver damage, inflammation, and fat accumulation. Chronic liver inflammation is one of the key factors leading to liver fibrosis and cirrhosis. By scavenging ROS, these compounds effectively reduce oxidative stress-induced liver damage. Tyr mitigates inflammation by inhibiting the activation of NF-κB and MAPK pathways, reducing the release of pro-inflammatory cytokines such as TNF-α and IL-6, thus protecting the liver from immune cell-induced damage. Additionally, Tyr lowers the expression of prooxidant enzyme NOX1 [187] as well as the mRNA expressions of transforming growth factor β1 (TGF-β1) and IL-6, reducing inflammation associated with non-alcoholic steatohepatitis (NASH) and showing protective effects against liver immune infiltration [188]. Studies have shown that the addition of Tyr to drinking water significantly alleviates chronic liver disease and liver fibrosis [189]. TH activates the Nrf2/ARE pathway, enhancing the synthesis of antioxidant enzymes and reducing ROS accumulation, thus preventing oxidative damage to liver cells. It also inhibits the activation of inflammation-related pathways such as NF-κB and COX-2, reducing chronic liver inflammation and significantly promoting liver protection [190]. Research indicates that 3,4-DHAP increases Nrf2 and HO-1 protein and mRNA expression, promotes Nrf2 nuclear translocation, and enhances autophagy, significantly reducing inflammation and indirectly contributing to liver protection [56].

#### 4.6.2. Improvement of Insulin Sensitivity and Regulation of Glucose Metabolism

Disorders in glucose metabolism, such as insulin resistance and elevated blood glucose, are major characteristics of diabetes. By promoting glucose metabolism and improving insulin sensitivity, these compounds can effectively improve blood sugar levels, aiding in the prevention and treatment of diabetes. In a streptozotocin (STZ)-induced diabetic rat model treated with Tyr, the compound significantly reduced the activity of 3-hydroxy-3-methylglutaryl-CoA reductase, exhibiting notable anti-inflammatory effects on the liver and pancreas [191,192], playing an important role in diabetes treatment. Specifically, Tyr improves insulin sensitivity by increasing glucose uptake and fatty acid metabolism, thereby alleviating symptoms of diabetes. TH promotes the expression of insulin and peroxiredoxin 6 (Prdx6) [112]. Its derivative, TH nicotinate (HT-N), significantly inhibits α-glucosidase activity, demonstrating strong antioxidant activity and anti-glycation properties, thus improving insulin sensitivity [193]. TH increases the activity of AMPK, enhances glucose metabolism, improves insulin sensitivity, lowers blood glucose levels, and improves glucose and insulin tolerance [194]. 3,4-DHAP regulates the AMPK pathway, enhancing insulin sensitivity, which further helps to improve blood glucose levels and alleviate insulin resistance. It also reduces serum triglycerides, cholesterol, malondialdehyde, and tumor necrosis factor α levels, further regulating metabolism [97].

#### 4.6.3. Anti-Fatty Accumulation and Improvement of Lipid Metabolism

Fat accumulation and lipid metabolism disorders, particularly in the liver and visceral organs, are major contributors to metabolic syndrome, obesity, and fatty liver disease [195]. Tyr intake can alleviate obesity and associated symptoms in high-fat diet (HFD)-fed mice by regulating thermogenesis mediated by peroxisome proliferator-activated receptor α (PPAR α) and the gut microbiota [196]. Additionally, TH dose-dependently inhibits intracellular triglyceride (TG) accumulation and the expression of lipogenesis stimulatory factors, promoting the lipolysis of primary visceral adipocytes and alleviating liver steatosis, demonstrating beneficial effects on obesity [197,198]. Studies show that TH alters the genes associated with adipocyte maturation and differentiation, inhibiting lipogenesis, including the synthesis of new fatty acids and cholesterol, thus benefiting hyperlipidemia [199,200]. TH regulates the transcription factors NF-κB, Nrf2, sterol regulatory element-binding protein 1c (SREBP-1c), and PPAR-γ, as well as their target genes, improving the function of white adipose tissue caused by HDL dietary intake in mice [198]. Furthermore, TH significantly improves liver steatosis and lipid deposition [201]. 3,4-DHAP not only helps maintain lipid homeostasis but also exerts positive metabolic regulatory effects by improving the balance of HDL function [202]. In disease models, 3,4-DHAP reduces total cholesterol, triglyceride, and low-density lipoprotein cholesterol levels in plasma and liver [203]. Research by Zhang Daijuan et al. suggests that 3,4-DHAP may improve pathological lipid metabolism by enhancing the activity of AMP-activated protein kinase (AMPK) and increasing the protein levels of phosphorylated sterol regulatory element-binding protein 1c (p-SREBP-1c) and phosphorylated acetyl-CoA carboxylase (p-ACC) [204].

## 5. Applications and Challenges for Phenolic Compounds

### 5.1. Application Prospects

Functional Foods and Health Products: Owing to their natural origin and wide-ranging health benefits, phenolic compounds have become ideal ingredients for developing functional foods and health supplements. Compounds like HT and 3,4-DHAP, endowed with potent antioxidant and anti-inflammatory properties, are already incorporated into various foods and beverages to enhance nutritional value and confer health effects. As technology advances, phenolic compounds will likely be more deeply integrated into daily diets and health products to help prevent chronic diseases and delay aging.

Drug Development and Adjunct Therapies: Phenolic compounds display remarkable pharmacological activities in anticancer, cardiovascular protection, and neuroprotection, offering promise for drug discovery. For instance, 3,4-DHAP, recognized for multiple anticancer mechanisms, is already considered a candidate for novel antitumor agents. Moreover, phenolic compounds may serve as adjuncts to chemo- and radiotherapy, potentially boosting efficacy and reducing side effects.

Chronic Disease Prevention: Thanks to their antioxidant, anti-inflammatory, and metabolic-regulating effects, phenolic compounds hold broad potential in preventing chronic diseases like diabetes, cardiovascular disorders, and neurodegenerative conditions. Ongoing research continues to underscore their significant role in managing chronic disease and safeguarding public health.

### 5.2. Bioavailability and Safety

Low bioavailability remains a major obstacle to phenolic compound utilization [205]. Due to their structural features, these compounds are poorly absorbed in the gastrointestinal tract and rapidly metabolized, restricting their in vivo activity [206]. Developing methods to enhance bioavailability—such as nano-drug delivery systems or other advanced transport technologies—will be crucial in future studies. Although phenolic compounds occur naturally in various foods and are associated with numerous health benefits, their safety in long-term, high-dose use requires further investigation.

HT is actively being developed as a potential supplement or preservative for the nutraceutical, cosmeceutical, and food industries. Toxicological assessments indicate a no-observed-adverse-effect-level (NOAEL) of 500 mg/kg/day [207]. Nanoparticle formulations (average size of ~260.90 nm) of 3,4-DHAP derivatives improve their oral bioavailability, offering new strategies to address the low bioavailability of phenolic drugs [208]. Ensuring safety and minimizing potential toxicity remain major challenges, especially when used at higher concentrations or as pharmaceutical agents [209,210].

The mechanisms of phenolic compounds’ bioactivity are diverse and complex. While substantial research has elucidated their antioxidant, anti-inflammatory, and metabolic-regulation effects, some of the key molecular signaling pathways remain incompletely understood. Further in-depth studies are necessary to clarify these compounds’ functions in different biological systems, thereby guiding more effective applications.

Moreover, phenolic compounds exhibit variable efficacy depending on their source, purity, extraction method, and mode of administration. Establishing unified quality standards and dosage protocols is essential for their clinical application [211] and for functional food development. Future work will focus on standardized extraction processes and product specifications to ensure consistency and effectiveness. By overcoming these hurdles, phenolic compounds will find broader applications in health promotion, facilitating innovation in functional foods, health products, and drug development for chronic disease prevention and treatment [212].

## 6. Conclusions and Outlook

In conclusion, tyrosol (Tyr), hydroxytyrosol (HT), and 3,4-Dihydroxyacetophenone (3,4-DHAP) are promising phenolic compounds with significant antioxidant, anti-inflammatory, anticancer, and cardiovascular protective properties. These compounds hold considerable potential for the prevention and treatment of chronic diseases, including cardiovascular conditions, neurodegenerative diseases, and cancer. Their mechanisms of action, such as free radical scavenging, oxidative stress reduction, and anti-inflammatory effects, highlight their therapeutic relevance.

However, challenges remain, particularly regarding their low bioavailability, which limits their clinical effectiveness. Advanced delivery systems, such as nanoparticle formulations or lipid-based carriers, are needed to enhance their absorption and bioavailability. Additionally, although these compounds show promise in short-term use, further research on their long-term safety, including toxicity and drug interactions, is essential before widespread clinical application.

Future research should focus on better understanding the molecular mechanisms underlying their therapeutic effects, including their influence on redox homeostasis, immune response, and cancer cell apoptosis. Furthermore, exploring combination therapies involving Tyr, HT, and 3,4-DHAP with conventional treatments may provide synergistic benefits, particularly for complex diseases like cancer and cardiovascular diseases.

In summary, while Tyr, HT, and 3,4-DHAP show great promise as therapeutic agents, overcoming challenges related to bioavailability, safety, and mechanistic understanding will be critical for their successful clinical application in treating chronic diseases.

## Data Availability

All data are contained within the article.
